# Pandemic Considerations on Essential Oral Health Care

**DOI:** 10.1177/0022034520979830

**Published:** 2020-12-09

**Authors:** H. Benzian, E. Beltrán-Aguilar, M.R. Mathur, R. Niederman

**Affiliations:** 1 Department Epidemiology and Health Promotion, WHO Collaborating Center Quality Improvement and Evidence-Based Dentistry, College of Dentistry, New York University, New York, NY, USA; 2 Global Health Center, Geneva Graduate Institute for Policy Studies, Geneva, Switzerland; 3 Department Epidemiology and Health Promotion, WHO Collaborating Center Quality Improvement and Evidence-Based Dentistry, College of Dentistry, New York University, New York, NY, USA; 4 Public Health Foundation of India, Gurugram, India; 5 Department of Public Health, Policy and Systems, University of Liverpool, Liverpool, UK; 6 Department Epidemiology and Health Promotion, WHO Collaborating Center Quality Improvement and Evidence-Based Dentistry, College of Dentistry, New York University, New York, NY, USA

**Keywords:** universal health coverage, dental health care, COVID-19, health care systems, dentistry organization & administration, dental care delivery

## Abstract

The coronavirus disease 2019 (COVID-19) pandemic revealed a lack of consensus on the concept of essential oral health care. We propose a definition of essential oral health care that includes urgent and basic oral health care to initiate a broader debate and stakeholder alignment. We argue that oral health care must be part of essential health care provided by any health system. Essential oral health care covers the most prevalent oral health problems through an agreed-on set of safe, quality, and cost-effective interventions at the individual and community level to promote and protect oral health, as well as prevent and treat common oral diseases, including appropriate rehabilitative services, thereby maintaining health, productivity, and quality of life. By default, essential oral health care does not include the full spectrum of possible interventions that contemporary dentistry can provide. On the basis of this definition, we conceptualize a layered model of essential oral health care that integrates urgent and basic oral health care, as well as advanced/specialist oral health care. Finally, we present 3 key reflections on the essentiality of oral health care. First, oral health care must be an integral component of a health care system’s essential services, and by implication, oral health care personnel are part of the essential health care workforce. Second, not all dental care is essential oral health care, and not all essential care is also urgent, particularly under the specific risk conditions of the pandemic. Third, there is a need for criteria, evidence, and consensus-building processes to define which dental interventions are to be included in which category of essential oral health care. All stakeholders, including the research, academic, and clinical communities, as well as professional organizations and civil society, need to tackle this aspect in a concerted effort. Such consensus will be crucial for dentistry in view of the Sustainable Development Goal’s push for universal health coverage, which must cover essential oral health care.

## COVID-19 Limiting Essential Health Care Needs

Is dental care an essential health care service? Every oral health care professional would likely respond with a resounding “yes.” However, what looks like a provocative question was put to a test during the peak of the coronavirus disease 2019 (COVID-19) pandemic. Receiving dental care was a challenge for many, not only those seeking regular dentist checkups and tooth-cleaning appointments but also for those with more serious oral health problems. The uncertainties around airborne virus transmission, the risks of potentially infectious aerosols from dental procedures, or even shortages of personal protective equipment (PPE) led regulating authorities around the world to issue guidance limiting or forbidding dental services. Consequently, many countries witnessed complete closures of public and private practices or service restrictions to the provision of emergency care. Patients with COVID-19 symptoms and dental problems were generally only admitted in designated hospitals or clinics with increased infection control capacities, such as negative pressure rooms (Clarkson et al. 2020; Occupational Safety and Health Administration [OSHA] and Department of Labor 2020).

Similar to other sectors of health care, terms like *essential, urgent*, and *emergency care* were used, assuming that there is an underlying consensus or common understanding of terminology. However, we think that agreement on the concepts and definition of essential care, including urgent and basic care, is missing in dentistry, particularly in the pandemic COVID-19 context.

## A Kerfuffle on Dental Care as an Essential Health Care Service

While many national authorities, the US Centers for Disease Control and Prevention (CDC) included, issued guidance documents for dental services and COVID-19 from later February 2020 onwards, the World Health Organization (WHO) published an Interim Guidance Note on “Considerations for the Provision of Essential Oral Health Services in the Context of COVID-19” in August 2020 (World Health Organization 2020). The WHO document summarizes service recommendations related to all aspects of dental services, including patient triage, operatory preparation, infection control, and selection of low-risk interventions, with the aim of supporting national service planning with the best possible protection of patients, providers, and public health in mind.

In the wake of the Interim Guidance’s release, a kerfuffle among key dental stakeholder organizations and the WHO ensued, sparked by misreporting from a major international news agency and the inclusion of preventive care as “non-essential health care.” The first report of Reuters stated that the WHO recommends foregoing any routine, nonessential dental care in a COVID-19 context. Subsequently, this reading was picked up by other media and stakeholders who were apparently not aware of the full WHO document. The American Dental Association (ADA) and other dental organizations rapidly issued statements to refute WHO’s alleged recommendation, stating that the provision of all dental care was essential and safe under all circumstances and that limiting dental services would lead to serious health and health system consequences.

WHO’s recommendation, however, advised to limit dental care to urgent or emergency care only in situations with high community spread, subject to national or local regulations reflecting the current epidemiology, infection risk, and its consequences in the health care system. The sentence in question read, “WHO advises that routine non-essential oral health care—which usually includes oral health check-ups, dental cleanings and preventive care—be delayed until there has been sufficient reduction in COVID-19 transmission rates from community transmission to cluster cases or according to official recommendations at national, sub-national or local level.” Only in such circumstances should routine care be postponed, a recommendation much in line with several national advisories, including the CDC recommendations from March 2020 (Centers for Disease Control and Prevention 2020). Moreover, the WHO Interim Guidance Note clearly emphasized the high importance of effective prevention during the COVID-19 pandemic but did not provide structured definitions of essential dental services. Unfortunately, a correction to the initial media release by the news agency a day later did not get the international attention that the first sensational headline generated. In the meantime, the word “non-essential” in the above sentence of the WHO Interim Guidance Note has actually been changed to “non-urgent.”

For the uninitiated observer, the controversy may just be the result of an unclearly worded guidance, sloppy journalistic coverage, or an increased media hyperbole for all things related to the COVID-19 pandemic. Yet, the debate revealed deeper and more far-reaching differences in fundamental concepts and understanding of what constitutes essential (oral) health care services and whether oral health care service interruptions are justified in the interest of public health.

## What Is Essential (Oral) Health Care?

The concept of essential (oral) health care needs to be considered on 2 levels: first, there is a macro level where oral health care is seen as part of an entire domain of other general health care services, such as in- and outpatient care, prescription drugs, pediatric care, and others (see Fig.). Second, as is the case with other medical specialties, within the domain of oral health care itself, there are interventions that can be defined as essential or advanced care.

**Figure.**
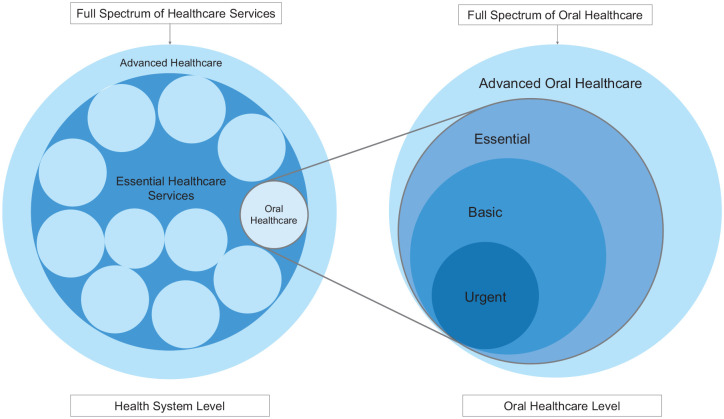
Layered concept of essential health care/oral health care.

Health is a fundamental element of human rights and, by implication, oral health as well. This is an important grounding for the concept that a certain set of health and public health services, universally available, is required for protection against common health risks and diseases (Eddy 1991). These are considered essential services for a dignified human existence. In most societies and health care systems, such services are public goods, meaning that there is a significant share of public or governmental responsibility to provide these services and to ensure equitable access by the entire population. When essential health and oral health care are not part of public health care systems or primary health care, appropriate financial protection should be available. Not having this set of essential health care services available would put people at physical, mental, and social harm or even risk of dying prematurely.

This is where the consensus oftentimes ends. The precise components of essential health care, the financing mechanisms, the definition of benefits, and the extent of private copayments are generally subject to a societal negotiation process. The resulting health care services and coverage are then reflecting historical heritage, social values and cultural traditions, and economic resources and capacities, but they also mirror the balance between needs and priorities, market or other power structures and civic participation, and the overall political and social governance. What is considered essential health care is therefore highly contextual and may vary widely within and between countries.

Decision makers and health planners do not automatically include oral health care when defining and designing essential health care services at a macro level. Instead, dental care is oftentimes seen as a separate, private responsibility with partial or full private financing (Wang et al. 2020). This occurs despite the major burden of oral diseases worldwide, their significant impact on health, well-being, and economies at large. The duality of theoretical inclusion and functional exclusion of dental care from the macro system creates an ethical dilemma and a double standard of essentiality. From the perspective of oral health professionals (and many health professionals and patients), as well as from a rights-based point of view, oral health care is seen as essential (hence with a strong element of public responsibility), yet health care systems are often deliberately not designed to provide equal access to oral health care for all, leaving coverage and financing to the individual’s private responsibility.

We argue that on a macro health care system level, oral health care should be an integral part of essential health care services. In fact, in many countries around the world, health systems cover oral health care and make no distinction between oral and general health care (Wang et al. 2020). However, which interventions are part of essential oral health care, prioritized or excluded varies greatly and is determined by national authorities, ideally with local needs and resources in mind.

## A Layered Concept of Essential Oral Health Care

One of the challenges when defining essential oral health care and prioritizing interventions is the lack of agreed criteria. The World Bank’s definition that “essential care comprises interventions that provide value for money, are implementable, and address substantial needs” provides some indication of critical aspects when selecting suitable interventions (Jamison et al. 2018).

As a starting point for further discussion and refinement, we propose the following working definition of essential oral health care:

Essential oral health care covers the most prevalent oral health problems through an agreed set of safe, quality, and cost-effective interventions at the individual and community level to promote and protect oral health, as well as prevent and treat common oral diseases, including appropriate rehabilitative services, thereby maintaining health, productivity, and quality of life.

By default, essential oral health care does not include the full spectrum of possible interventions that contemporary dentistry can provide. On the basis of this working definition, we conceptualize a layered model of essential oral health care that integrates various related terms used in discussions around COVID-19 and universal health coverage (UHC) but were at times poorly defined (Fig.).

In this concept, the outer circle comprises the full spectrum of contemporary oral health care with every possible intervention, from simple tooth extractions to advanced or specialist care. Elective interventions that are part of cosmetic or esthetic dentistry or more elaborate variants of simpler options, those lacking a solid evidence base, or those that address rare conditions are part of the outer layer of the model. Essential oral health care is a subset of this full basket of interventions and services, and urgent oral health care a further subset within essential oral health care.

Within essential oral health care, the group of emergency/urgent interventions is the one with a generally agreed definition. As part of the COVID-19 measures, the ADA stated that “dental emergencies are potentially life threatening and require immediate treatment to stop ongoing tissue bleeding, alleviate severe pain or infection” (American Dental Association 2020b) The ADA’s guidance also introduced the concept of urgent care: “Urgent dental care focuses on the management of conditions that require immediate attention to relieve severe pain and/or risk of infection and to alleviate the burden on hospital emergency departments. These should be treated as minimally invasively as possible” (American Dental Association 2020b).

We propose framing these 2 concepts more broadly, given that dentists are generally referring patients in life-threatening situations to specialist or emergency hospital care. The following proposal merges the ADA’s definitions for urgent and emergency care:

Urgent oral health care describes interventions for oral diseases and conditions that are serious in terms of bleeding, infection, swelling, or pain or that otherwise impact with significant consequences if left unattended, therefore requiring treatment or referral without delay.

This combination simplifies the concept and makes it easier to understand for policy planners seeking a basis for developing service guidance. Urgent oral health care procedures are therefore the smallest group, yet the most important to avoid serious complications.

Within the layered model of essential oral health care, we include the idea of basic oral health care. Under ideal conditions, basic oral health care would be equal to essential care with a health care system covering all essential services (Tomar and Cohen 2010). Since most health systems are constrained by limited financing, workforce, or other resources, a selected subset of essential oral health care may be prioritized for implementation, thereby maximizing the available resources and health outcomes. Basic oral health care comprises our definition of urgent care as nonnegotiable and also covers other elements of essential care, including nonurgent preventive services. The WHO’s Basic Package of Oral Care is a prime example of such a bundle, covering elements of urgent care, simple (aerosol-free) restorative care, and prevention (Frencken et al. 2002). Since its publication in 2002, thinking and development have evolved. Thus, we propose to define basic oral health care as follows:

Basic oral health care describes a minimum subset of essential oral health care services that are universally available to everyone in a given population, regardless of the ability to pay. Included quality services are safe, prioritize the most frequent diseases and conditions with best health outcomes at the lowest cost, and can be provided for everyone with the resources available.

The interventions included in such a minimum package of basic oral health care need to be determined locally, taking into account community and population needs, the burden of disease and priorities, the available resources of the health care system, financing priorities, and the political support and societal or cultural priorities. It should be noted that the term *basic* does not refer to interventions of lower quality but rather to those that are considered an essential minimum in terms of service coverage. At the same time, “advanced” or “specialist” services do not carry the connotation of higher quality but rather indicate that these services are not part of essential services; some elective interventions may even be seen as nonessential.

As much as the elements comprised in basic care need to be determined locally, the same applies to the care included in the essential oral health care domain. A rich country with a mature health system, or following society demand and consensus, may include aspects of advanced or specialist care in their essential care package (such as orthodontics for children). For other countries with constraints in terms of capacities and resources, even offering simple fillings may be a significant inclusion in their basic oral health care package. The benefit of a layered concept is that it allows for local care “titration” on the continuum from urgent to specialist care, allowing for categorization and prioritization with available resources and needs in mind. This approach aligns with the conceptual thinking around primary oral health care, where the most frequent demand is covered by essential services (Watt et al. 2019).

The COVID-19 pandemic highlights the challenges of adapting oral health care to diseases with airborne transmission and significant knowledge gaps related to risk (Beltrán-Aguilar et al. 2021). In applying the proposed definition of basic oral health care (safe, cost-effective, addressing most frequent needs, universally available) to the COVID-19 context, we propose that basic oral health care is so safe, efficient, and cost-effective that it can be provided universally, irrespective of whether there is a public health emergency or not (Benzian and Niederman 2020; Cothron and McLeod 2020). This moves the discussion from “why” to “how,” and the question of whether or not oral health care can be provided relies entirely on the epidemiological status of the public health emergency and the guidance of public health authorities.

## Three Lessons about Essentiality of Oral Health Care from COVID-19

Circling back to the starting point of the article, 3 key lessons can be drawn from the COVID-19 pandemic in relation to dentistry. First, oral health care must be an integral component of the essential health care system’s services, and by implication, oral health care personnel are part of essential health care workforce. This is an important consideration in view of access to a future COVID-19 vaccine, PPE for clinical care, and even economic support measures by governments (American Dental Association 2020a; National Academies of Sciences 2020). This insight, however, also places ethical and political responsibilities on stakeholders, including governments, dental associations, employers, and the insurance industry, to make sure that what is deemed essential is also available, accessible, and affordable for everyone, especially under pandemic conditions of risk and increased numbers of people without dental health insurance. The service limitations or loss of dental insurance coverage experienced during the height of the COVID-19 pandemic gave many people an idea of the challenges and hardships that millions of people are facing when it comes to accessing dental care in times without a pandemic.

Second, it must be clarified and acknowledged that not all dental care is essential, and not all essential care is also urgent, particularly under the specific risk conditions of the pandemic. This facilitates the required prioritization of certain oral health care interventions in the context of a public health emergency and facilitates the inclusion of essential oral health care into general health care. At least some of the kerfuffle regarding oral health care during the COVID-19 pandemic noted above might have been averted with such clear differentiation between urgent and nonurgent essential oral health care. Prevention of oral diseases is essential, although, using our definition, not urgent oral health care. However, whether different preventive care approaches would be essential or not will depend on the scientific evidence supporting their efficacy. Mindful use of language around essential oral health care reduces misunderstandings.

The third and last lesson relates to the need for criteria, evidence, and consensus processes to define which dental interventions are to be included in which category of essential oral health care. This is a formidable and highly pertinent challenge for all stakeholders, including the research, academic, and clinical communities (Norheim 2017). The ethical driver of such a process should be the immense unmet oral health needs across populations in all countries, and political decisions need to be grounded in science and evidence.

## Considerations for Integration and UHC

UHC is part of the health-related goal 3 of the United Nations’ Sustainable Development Goals, which have been unanimously adopted by all nations, providing a framework for national and international development until 2030. Oral health was recently included in the political commitment to strengthen UHC (United Nations General Assembly 2019). Defining essential oral health care for UHC is critical to model costs and financing, as well as develop options for insurance coverage, all key prerequisites for political decisions in the context of priority setting (Chalkidou et al. 2016).

All of this needs forward-looking leadership among all stakeholder groups, including civil society, to initiate and constructively advance the discussion around essential oral health care and making it universally available for everyone.

## Author Contributions

H. Benzian, E. Beltrán-Aguilar, R. Niederman, contributed to conception and design, drafted and critically revised the manuscript; M.R. Mathur, contributed to conception, critically revised the manuscript. All authors gave final approval and agree to be accountable for all aspects of the work.
